# The type of anticoagulant used for plasma collection affects in vitro* Rhodococcus equi* assays

**DOI:** 10.1186/s13104-022-05933-4

**Published:** 2022-02-14

**Authors:** Alejandra A. Rivolta, Dana C. Pittman, Amanda J. Kappes, Robert K. Stancil, Clark Kogan, Macarena G. Sanz

**Affiliations:** 1 Department of Veterinary Clinical Sciences, College of Veterinary Medicine, Washington State University, Pullman, WA USA; 2 Department of Mathematics, Washington State University, Pullman, WA USA

**Keywords:** *Rhodococcus equi*, *Prescotella equi*, Anticoagulants, Plasma, EDTA, Citrate, Macrophages

## Abstract

**Objective:**

The efficacy of *Rhodococcus equi*-specific hyperimmune plasma (HIP) is usually evaluated in vitro. Anticoagulants (AC) used for plasma collection can negatively impact bacterial replication but their effect on *R. equi* growth has not been evaluated. The aim of this study was to establish the effect that AC routinely used in veterinary medicine (ACD, K_2_EDTA, Li Heparin, and Na Citrate) have on in vitro* R. equi* growth. To assess this, in vitro assays commonly used to test HIP efficacy (direct effect on microorganism and macrophage infection), were performed using each AC and non-treated bacteria.

**Results:**

There was no direct effect of ACD, Li Heparin or Na Citrate on *R. equi* growth. These AC significantly (p < 0.05) delayed growth for 12 h following opsonization. The number of *R. equi* colonies after macrophage infection was significantly (p < 0.05) lower 72 h post-opsonization with Na Citrate. K_2_EDTA inhibited the formation of *R. equi* colonies by 12 h in all the assays. In conclusion, AC should be taken into consideration when interpreting in vitro results as their negative effect on bacterial growth may be mistakenly interpreted as HIP efficacy. ACD and Li Heparin appear more appropriate for the selected assays.

**Supplementary Information:**

The online version contains supplementary material available at 10.1186/s13104-022-05933-4.

## Introduction

*Rhodococcus equi* (*R. equi*) is a Gram-positive bacterium that infects macrophages and causes bronchopneumonia [1, 2] and extrapulmonary disease [3, 4] in young foals worldwide. There is no vaccine against this condition; therefore, *R. equi*-specific hyperimmune plasma of equine origin (HIP) is administered to neonatal foals for prophylaxis. However, the protective mechanism of HIP is poorly understood [5–7]. The in vivo study of HIP is complicated by the fact that animals such as mice [8] and guinea pigs [9] don’t develop typical lesions after experimental infection. As a result, in vitro assays are used to investigate the effect that HIP has on *R. equi* infection of macrophages [10, 11].

Multiple in vitro studies have evaluated the effect that HIP has on *R. equi* intracellular survival [10, 12, 13]; however, little attention has been paid to the type of anticoagulant (AC) used to collect these plasma products. Sodium citrate (Na Citrate) is commonly used to collect large volumes of plasma but other AC such as acid citrate dextrose (ACD), ethylenediaminetetraacetic acid (EDTA), and lithium heparin (Li Heparin) are also used. These compounds prevent blood coagulation by different mechanisms and in addition, some directly compromise in vitro growth of microorganisms [14–16]. However, the effect that AC may have on *R. equi *in vitro assays has not been evaluated. Failure to recognize these effects can lead to misinterpretation of the data and result in inappropriate estimation of product efficacy [14] jeopardizing patient safety. The objective of this study was to evaluate the effect that routinely used AC namely ACD, K_2_EDTA, Li Heparin and Na Citrate have on commonly performed *R. equi *in vitro assays.

## Main text

### Methods

#### Bacterial strain and growth

Pathogenic *R. equi* #ATCC 103^+^ expressing *gfp* gene [17] was cultured from glycerol stock on brain heart infusion (BHI; BD Difco, MD) agar plates at 37 °C for 48 h. A colony was inoculated in 5 mL of BHI following incubation (37 °C for 18 h). Concentration was estimated using optical density and confirmed by dilution plating and colony forming unit (CFU) counts after 48 h.

#### Cell culture

Murine macrophages RAW264.7 (ATCC TIB-71, MD) were irradiated as previously described [18] to prevent cell multiplication. Cells were maintained in Dulbecco's Modified Eagle Medium (DMEM; Gibco, NY) containing 10% fetal bovine serum, and 1% penicillin–streptomycin (Sigma Aldrich, MO).

#### Anticoagulant effect on R. equi growth

Media (BHI) was added to Vacutainer tubes® (BD, NJ) containing K_2_EDTA, Li Heparin, Na Citrate or ACD (1:7 AC:BHI). Thereafter, 0.5 mL of BHI-AC was mixed with 1.5 mL of BHI containing 1–3 × 10^4^ CFU of *R. equi*. Tubes were incubated at 37 ºC and CFU/mL were calculated at 0, 8, 12, 18, 24, and 32 h post-incubation (time to stationary phase) by serial plate dilution. *R. equi* was also grown in BHI without AC (positive control) and in BHI/PBS (1:7) to account for dilution.

#### Plasma effect on R. equi growth

Plasma from two healthy horses from the Washington State University research herd was collected using Vacutainer® tubes containing the AC above mentioned. Serum was also collected. Briefly, whole blood was collected in the tubes and centrifuged at 500×g 15 min to allow separation of the plasma/serum from the red blood cells. Plasma/serum (0.5 mL) from each horse was mixed with 1.5 mL of BHI containing 1–3 × 10^4^ CFU *R. equi*. Tubes were incubated at 37 ºC and CFU/mL were calculated at 0, 6, 12, 18, 24, and 32 h post-incubation using serial dilution.

#### R. equi opsonization

Opsonization was performed as previously described [19]. Briefly, plasma and serum from 3 healthy horses from the WSU research herd was collected as described above and pooled. Each plasma/serum was mixed with BHI containing 1–3 × 10^6^ CFU/mL of *R. equi* (v/v 1:3) for 30 min at 37 °C 60 rpm. Thereafter, bacteria were washed with PBS, resuspended in phagocytic media, and used for intracellular infections. Non-opsonized *R. equi* that underwent the same manipulation was also included.

#### Intracellular assay

Infections were performed as described before [10] with modifications. Briefly, RAW264.7 monolayers (1 × 10^5^ cells/well) were incubated overnight on 24-well tissue culture plates (Eppendorf, Germany) and washed with warm PBS prior to infection. Phagocytic buffer containing opsonized or non-opsonized *R. equi* at multiplicity of infection of 20 was added to each well and cells were incubated 1 h at 37 °C. Monolayers were then washed to remove unbound bacteria; and incubated 30 min for bound bacteria to be internalized. Media was replaced with complete DMEM (cDMEM) with 20 μg/mL of amikacin sulfate for 1 h to kill extracellular bacteria. Cells were then washed and incubated in cDMEM until lysis at 0, 24, 48, and 72 h post-infection and detachment for fluorescence microscopy (24 h). Lysis was achieved using saponin 0.1%, scraping, microtube homogenizer and centrifugation (10,000×*g* 10 min). Bacterial growth was determined by dilution plating of lysates.

#### Fluorescence microscopy

Infected cells were detached using cell dissociation buffer (Thermo Fisher, MA) immediately (T_0_) and 24 h after infection. Cells were stained with ProLong™ Gold-Antifade-Mountant with DAPI (Invitrogen, CA). Three hundred macrophages were counted and the number of infected cells and cells with 10 or more bacteria were established using the ImageJ software (NIH, Bethsda, MD) as described before [10].

#### Statistical analysis

Data were analyzed using R studio statistical software (https://www.r-project.org/). Normality and variance were assessed with Shapiro-Wilks and Levene’s tests respectively. For CFU/mL data analysis was log transformed. Changes in CFU/mL for BHI and plasma assays were evaluated using repeated measures two-factor analysis of variance (ANOVA) with interaction using a random effect. Changes in intracellular CFU/mL were evaluated using repeated measures two-factor ANOVA. Post-Hoc tests were conducted using the Dunnett’s test. The experiments were performed in duplicates and repeated on 3 different days. Significance was set up at p < 0.05.

### Results

#### Anticoagulant effect on R. equi growth

**Fig. 1:**
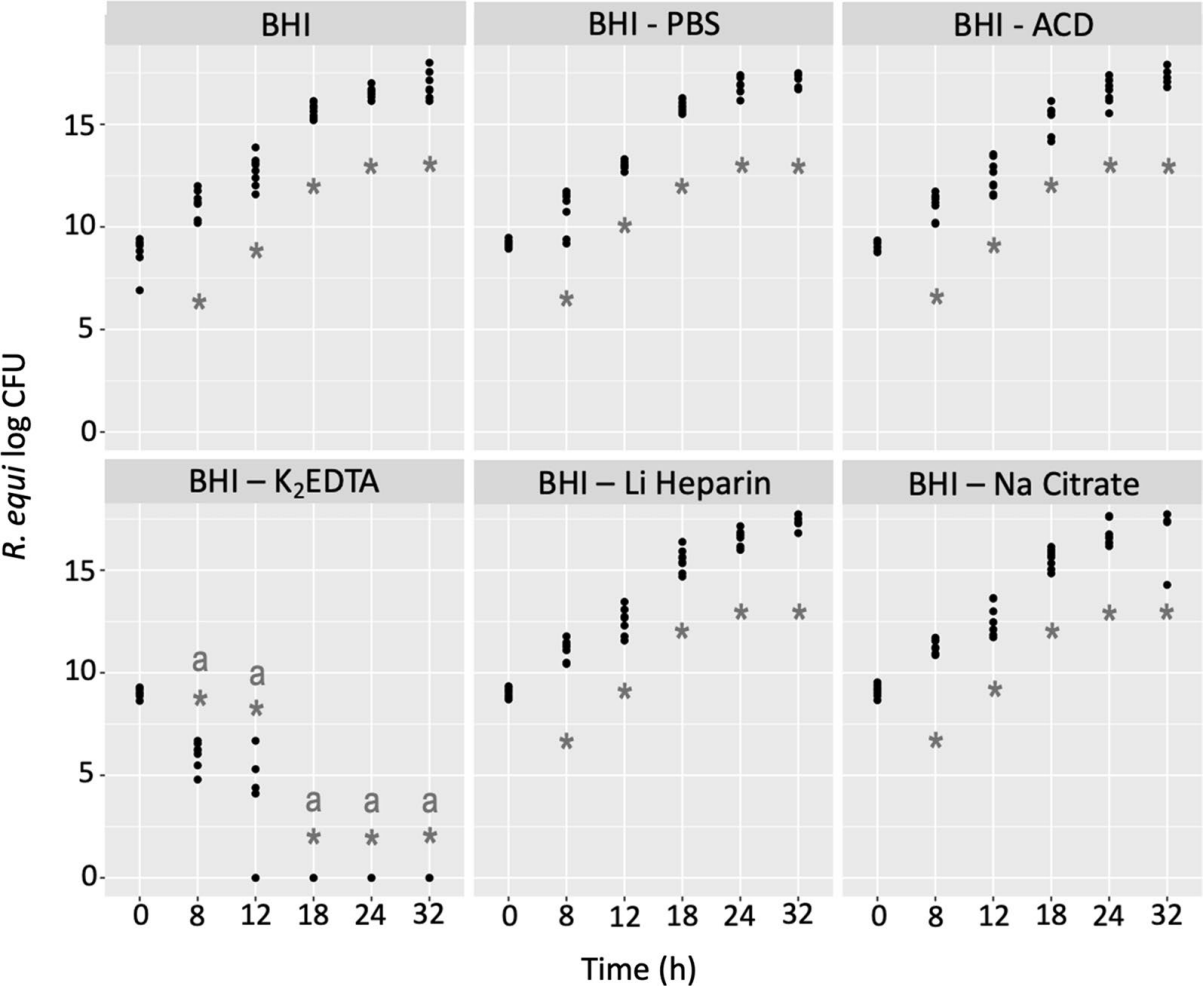
*R. equi* (log CFU/mL) after 0.5 mL of plasma collected with different AC (ACD, K_2_EDTA, Li Heparin, Na Citrate) was mixed with 1.5 mL of BHI containing 1–3 × 10^4^ CFU of *R. equi* for 32 h at 37 ºC. CFU/mL were counted immediately after mixing (T_0_) and 8, 12, 18, 24, and 32 h thereafter. BHI, and BHI/PBS were added as controls. Asterisks (*) indicate significant change in CFU/mL from T_0_ (p < 0.001) by group. Letters indicates significant (p < 0.001) differences in CFU/mL between groups at a specific timepoint

*R. equi* concentration was not different between groups at T_0_. CFU/mL significantly (p < 0.001) increased 8 h post-inoculation and at every timepoint thereafter in BHI and BHI/PBS. Similar growth overtime was observed in tubes with ACD, Li Heparin and Na Citrate. No significant differences were observed in the number of CFU/mL between these groups at any time. In contrast, *R. equi* cultured in K_2_EDTA tubes had significantly (p < 0.001) lower CFU/mL by 8 h post-inoculation. Moreover, there were no visible CFU by 18 h post-inoculation; thus, CFU/mL were significantly (p < 0.001) lower than the rest of the groups at all time points thereafter (Fig. 1)*.*

#### Plasma effect on R. equi growth

**Fig. 2:**
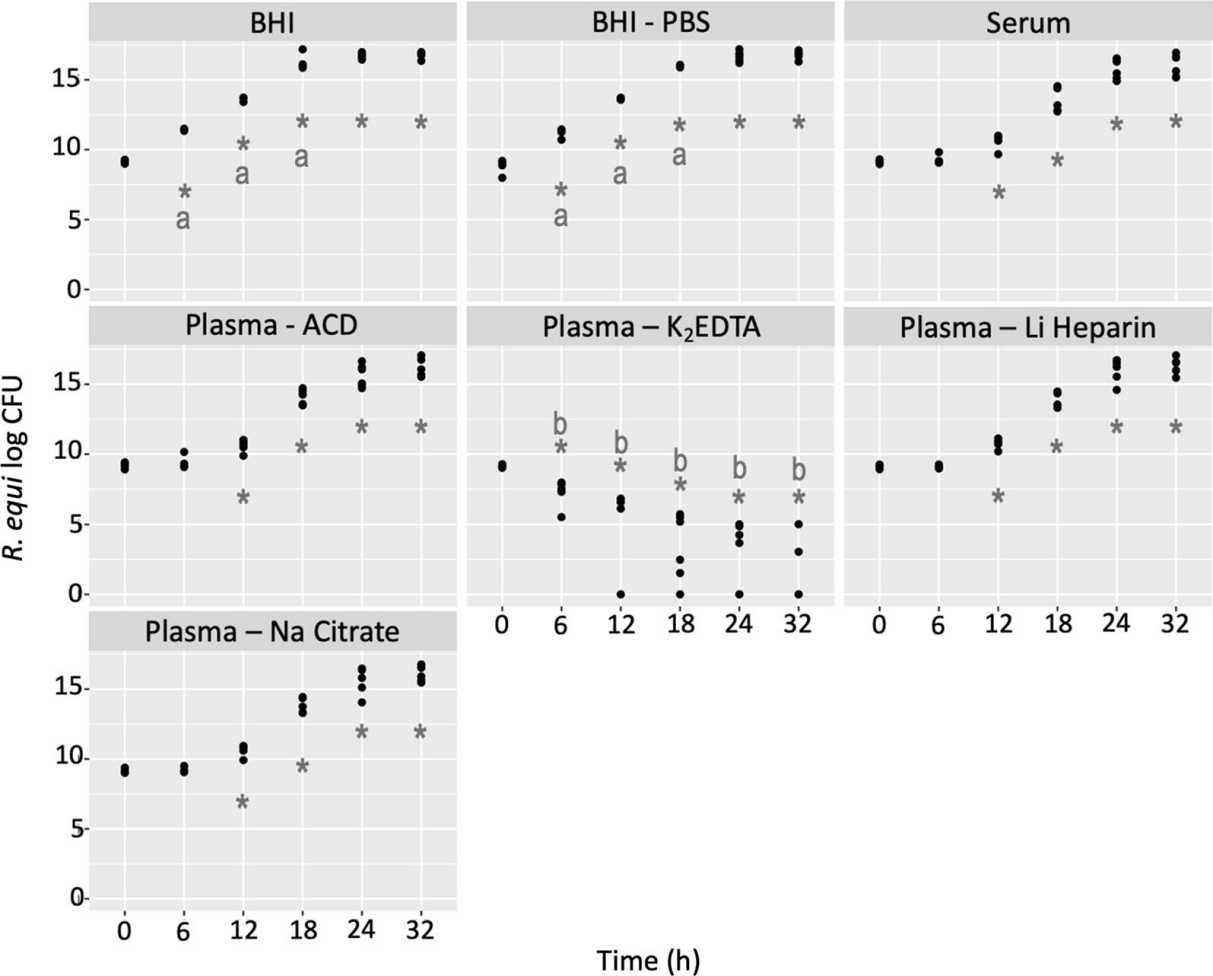
*R. equi* (log CFU/mL) after 0.5 mL of serum or plasma collected with different AC (ACD, K_2_EDTA, Li Heparin, and Na Citrate) were mixed with 1.5 mL of BHI containing 1–3 × 10^4^ CFU/mL of *R. equi* for 32 h at 37 ºC. CFU/mL were counted immediately after mixing (T_0_) and 6, 12, 18, 24, and 32 h thereafter. BHI, and BHI/PBS were added as controls. Asterisks (*) indicate significant change in CFU/mL from T_0_ (p < 0.05) by group. Different letters denote significant (p < 0.05) differences in CFU/mL between groups at a specific timepoint

*R. equi* CFU/mL significantly (p < 0.001) increased by 6 h post-inoculation in BHI and BHI/PBS. *R. equi* cultured in serum or plasma collected using ACD, Li Heparin, and Na Citrate grew significantly (p < 0.05) at every time point from 12 h post-inoculation. There was no significant difference in CFU/mL between these groups at any time. Tubes incubated with plasma containing K_2_EDTA had significantly (p < 0.001) lower CFU/mL by 6 h post-inoculation and had no visible CFU by 18 h post-inoculation. Thus, CFU/mL were significantly (p < 0.001) lower than the rest of the groups at all time points thereafter (Fig. 2).

#### Intracellular assay

**Fig. 3:**
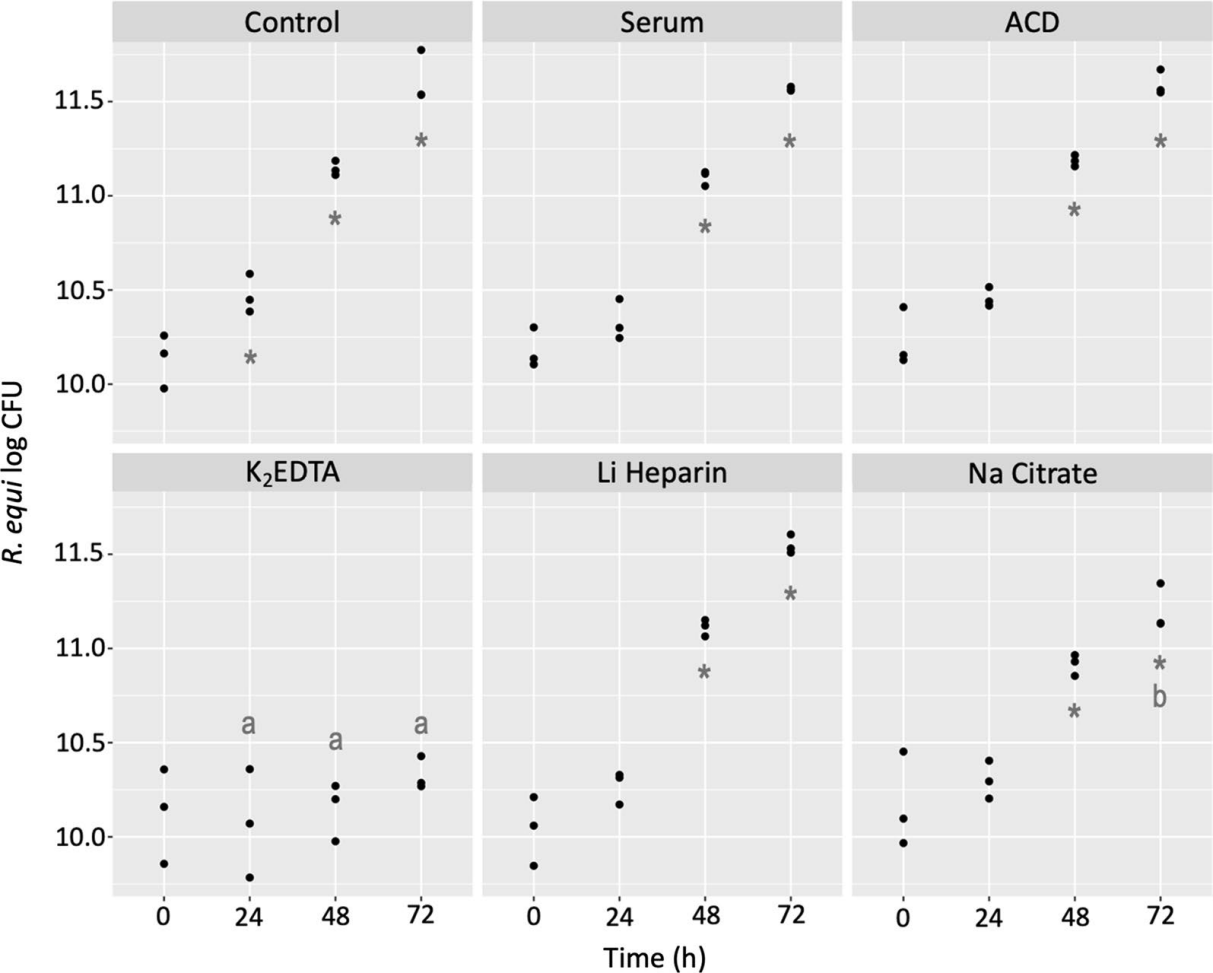
*R. equi* (log CFU/mL) after RAW264.7 cells were infected with non-opsonized (control) or opsonized (serum or plasma collected with ACD, K_2_EDTA, Li Heparin, or Na Citrate) bacteria and lysed at 0, 24, 48, and 72 h post-infection. Asterisks (*) indicate significant growths from T_0_ (p < 0.05). Different letters denote significant (p < 0.05) differences in CFU/mL between groups at a specific timepoint

At T_0_, there were no differences in CFU/mL between groups. Non-opsonized *R. equi* (CFU/mL) grew significantly (p = 0.0082) overtime inside macrophages after 24 h post-infection. While *R. equi* opsonized with serum or plasma collected with ACD, Li Heparin, and Na Citrate, grew significantly (p < 0.001) inside macrophages by 48 and 72 h post-infection; growth was significantly (p = 0.02) lower in Na Citrate by 72 h. In contrast, bacteria opsonized with K_2_EDTA displayed no growth overtime inside macrophages at any of the time points evaluated. Therefore, CFU/mL were significantly (p < 0.001) lower in K_2_EDTA than in the other groups at 24, 48, and 72 h post-infection (Fig. 3).

There were no significant differences in the number of infected cells per 300 macrophages or in the number of macrophages infected with 10 or more *R. equi* at T_0_. *R. equi* opsonized with plasma collected with K_2_EDTA had significantly (p = 0.04) fewer macrophages infected with 10 or more bacteria 24 h post-inoculation.

### Discussion

This study shows that the choice of AC significantly influences the results of the selected in vitro assays which are commonly used to study *R. equi* infection and the efficacy of HIP (Additional file 1: Figs. S1 and S2) [10–19, 23]. Overall, exposure of *R. equi* to K_2_EDTA resulted in inhibition of CFU formation shortly after exposure; this was not observed when other AC were used. This is important as growth inhibition of *R. equi* is a desirable effect of HIP. Most of the published work evaluating in vitro* R. equi* growth does not report the type of AC used for plasma collection. Therefore, it is difficult to establish if the effect of the AC was taken into consideration at the time of results interpretation. Failure to do so has led to equivocal efficacy reports in humans [14]. ACD and heparin did not have any significant impact on any of the assays evaluated.

As described for other Gram-negative and positive bacteria [14, 15, 20, 21], direct exposure to K_2_EDTA in broth resulted in complete inhibition of CFU by 18 h. This is likely the result of the strong Ca^2+^ and Mg^2+^ chelating capacity of K_2_EDTA increasing cell permeability and fragility which may lead to cell lysis [16]. Interestingly, a similar effect was not observed when the other citrate-based AC (Na Citrate and ACD) were used. Citrate-based anticoagulants prevent coagulation by chelating ionized calcium present in the blood to form non-ionized calcium-citrate complexes; however, their chelating effect is weaker than that of K_2_EDTA, especially for Mg^2+^ [15, 22]. Heparin, an AC that inhibits coagulation mainly by enhancing the activity of antithrombin III, didn’t show a direct effect on *R. equi* growth. This was expected as the authors could find no evidence that antithrombin is relevant to bacterial survival.

In addition to the effects seen with K_2_EDTA, there was a bacteriostatic effect observed the first 12 h post-inoculation with the other plasmas and serum. This is likely the effect of the antimicrobial proteins normally present in these bodily fluids as serum was collected without AC [23].

Intracellular infections were performed using murine macrophages which have been used to study *R. equi* infection [10, 13, 24]. Opsonization of *R. equi* with *R. equi*-specific antibodies increases microorganism uptake by Fcγ receptors of macrophages and enhances their oxidative burst [19, 25, 26]. Moreover, plasma and serum boost *R. equi* killing by enhancing phagosome-lysosome fusion [27]. Na Citrate significantly decreased intracellular *R. equi* growth overtime suggesting that sufficient chelation of calcium to weaken cell wall occurs [16], although other mechanism of growth inhibition such as hyperosmolarity of the solution [28, 29] and partial complement inhibition [15] can’t be ruled out. Interestingly, opsonization with plasma collected with K_2_EDTA resulted in intracellular *R. equi* growth inhibition but not death in the 72 h period studied. EDTA partially decreased bacterial deposition of C5b9, a multimer that mediates bacterial killing on *Neisseria meningitidis* [15]. Others have shown that EDTA inhibited CR3-mediated binding on RAW264.7 cells, decreasing phagocytosis of the Gram-negative bacterium *Borrelia burgdorferi* [30]. The exact mechanism for the lack of *R. equi* death inside macrophages in our study remains to be determined.

There were no significant differences in the number of infected cells per 300 macrophages or number of macrophages with 10 or more *R. equi* immediately post-infection suggesting that AC do not affect the initial *R. equi* uptake by macrophages. The number of macrophages containing 10 or more bacteria after 24 h was significantly lower when EDTA was used. This likely reflects the direct effect of K_2_EDTA on *R. equi* growth. Unfortunately, bacterial fluorescence was not reliable past 24 h (data not shown) which limited our ability to evaluate cell replication past this point [31]. Thus, only CFU data are reported for subsequent timepoints.

### Conclusion

Anticoagulants significantly influenced the selected *R. equi *in vitro assays. Specifically, K_2_EDTA inhibited CFU formation and resulted in intracellular growth inhibition, whereas Na Citrate delayed intracellular growth. Failure to recognize these effects can lead to misinterpretation of the data and inappropriate estimation of product efficacy. The use of ACD and Li Heparin appears to be more appropriate choices for the selected in vitro assays.

## Limitations

- Only one strain has been used for this study (*R. equi* 103 s-gfp).

## Supplementary Information

**Additional file 1: Figure S1.** *R. equi* (log CFU/mL) after either 0.5 mL of *R. equi*-specific HIP (HIP-Re) or hyperimmune plasma not specific for *R. equi* (HIP-NoRe) were mixed with 1.5 mL of BHI containing 1-3 × 10^4^ CFU/mL of *R. equi *for 36 h at 37 °C. All plasmas were collected using sodium citrate. CFU/mL were counted immediately after mixing (T0) and 12, 18 and 24 h thereafter. BHI was added as control. Statistical significance between groups is noted in the graph. **Figure S2.** *R. equi* (log CFU/mL) after 1x10^5^ RAW264.7 cells were infected with non-opsonized (control) or opsonized with *R. equi*-specific HIP (HIP-Re) or serum *R. equi* at a MOI 20 for 1 h. Infected cells were lysed at 0, 24, 48, and 72 h post-infection, were plated in BHI agar plates and bacterial colonies were counted 48 h later. Statistical significance between groups is noted in the graph.

## Data Availability

Data is available upon request. Please contact Dr Macarena G. Sanz (macarena@wsu.edu).

## Abbreviations

AC: Anticoagulant
ACD: Acid citrate dextrose
ANOVA: Analysis of variance
cDMEM: Complete DMEM
CFU: Colony forming units
EDTA: Ethylenediaminetetraacetic acid
HIP: *Rhodococcus equi-* Hyperimmune plasma
Li Heparin: Lithium heparin
Na Citrate: Sodium citrate
*R. equi*: *Rhodococcus equi*
