# Supplementary material for: The type of anticoagulant used for plasma collection affects in vitro Rhodococcus equi assays
Source: BMC Res Notes. 2022 Feb 14;15:50. doi: 10.1186/s13104-022-05933-4 (PMC8842809; doi:10.1186/s13104-022-05933-4)
Supplement: Supplementary file 1 — Additional file 1: Figure S1. R. equi (log CFU/mL) after either 0.5 mL of R. equi-specific HIP (HIP-Re) or hyperimmune plasma not specific for R. equi (HIP-NoRe) were mixed with 1.5 mL of BHI containing 1-3 × 10^4^ CFU/mL of R. equi for 36 h at 37 °C. All plasmas were collected using sodium citrate. CFU/mL were counted immediately after mixing (T0) and 12, 18 and 24 h thereafter. BHI was added as control. Statistical significance between groups is noted in the graph. Figure S2. R. equi (log CFU/mL) after 1x10^5^ RAW264.7 cells were infected with non-opsonized (control) or opsonized with R. equi-specific HIP (HIP-Re) or serum R. equi at a MOI 20 for 1 h. Infected cells were lysed at 0, 24, 48, and 72 h post-infection, were plated in BHI agar plates and bacterial colonies were counted 48 h later. Statistical significance between groups is noted in the graph. [file 13104_2022_5933_MOESM1_ESM.docx]

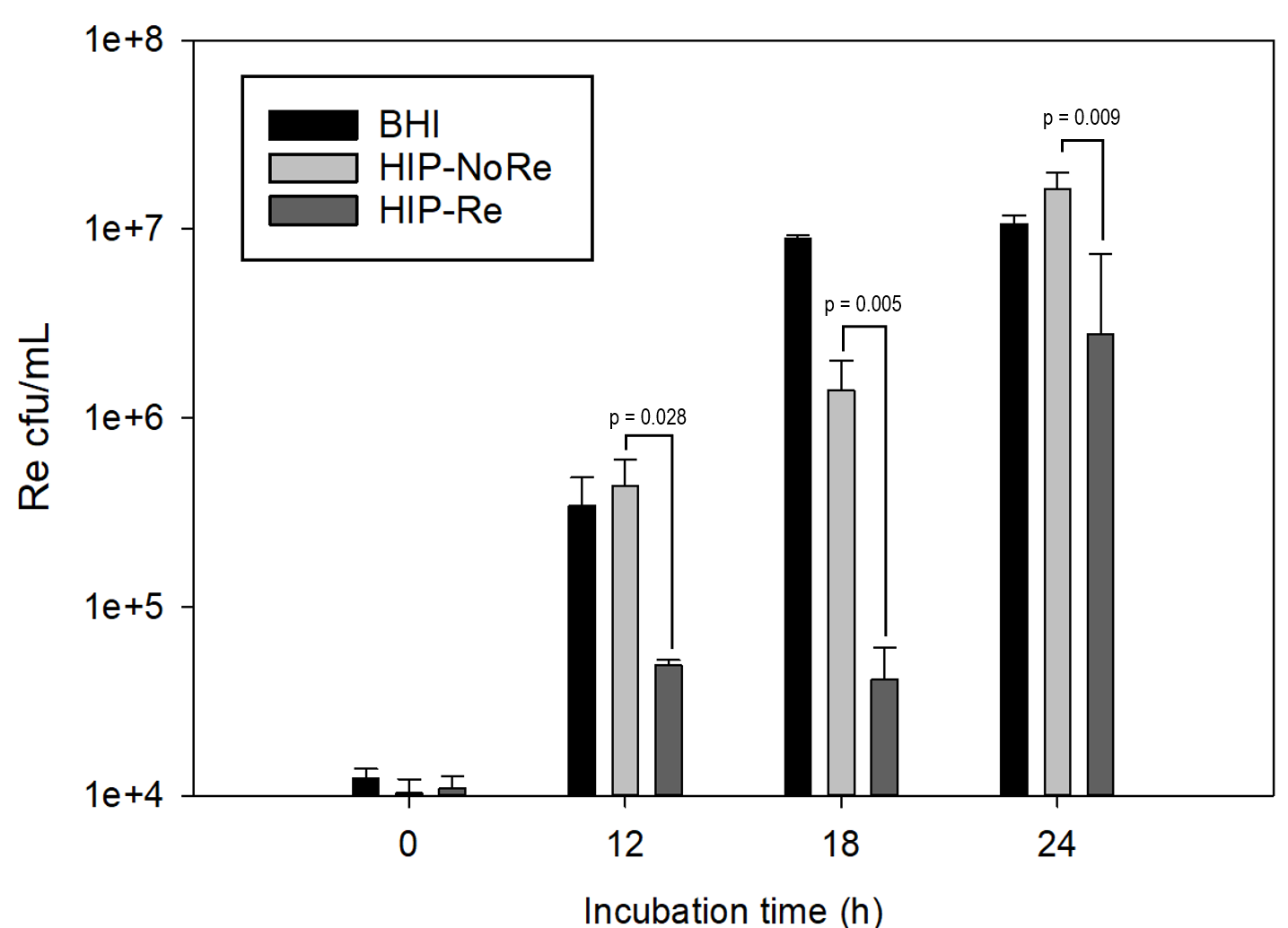


**Figure S1.** *R. equi* (log CFU/mL) after either 0.5 mL of *R. equi*-specific HIP (HIP-Re) or hyperimmune plasma not specific for *R. equi* (HIP-NoRe) were mixed with 1.5 mL of BHI containing 1-3 x 10^4^ CFU/mL of *R. equi* for 36 h at 37 ºC. All plasmas were collected using sodium citrate. CFU/mL were counted immediately after mixing (T0) and 12, 18 and 24 h thereafter. BHI was added as control. Statistical significance between groups is noted in the graph.


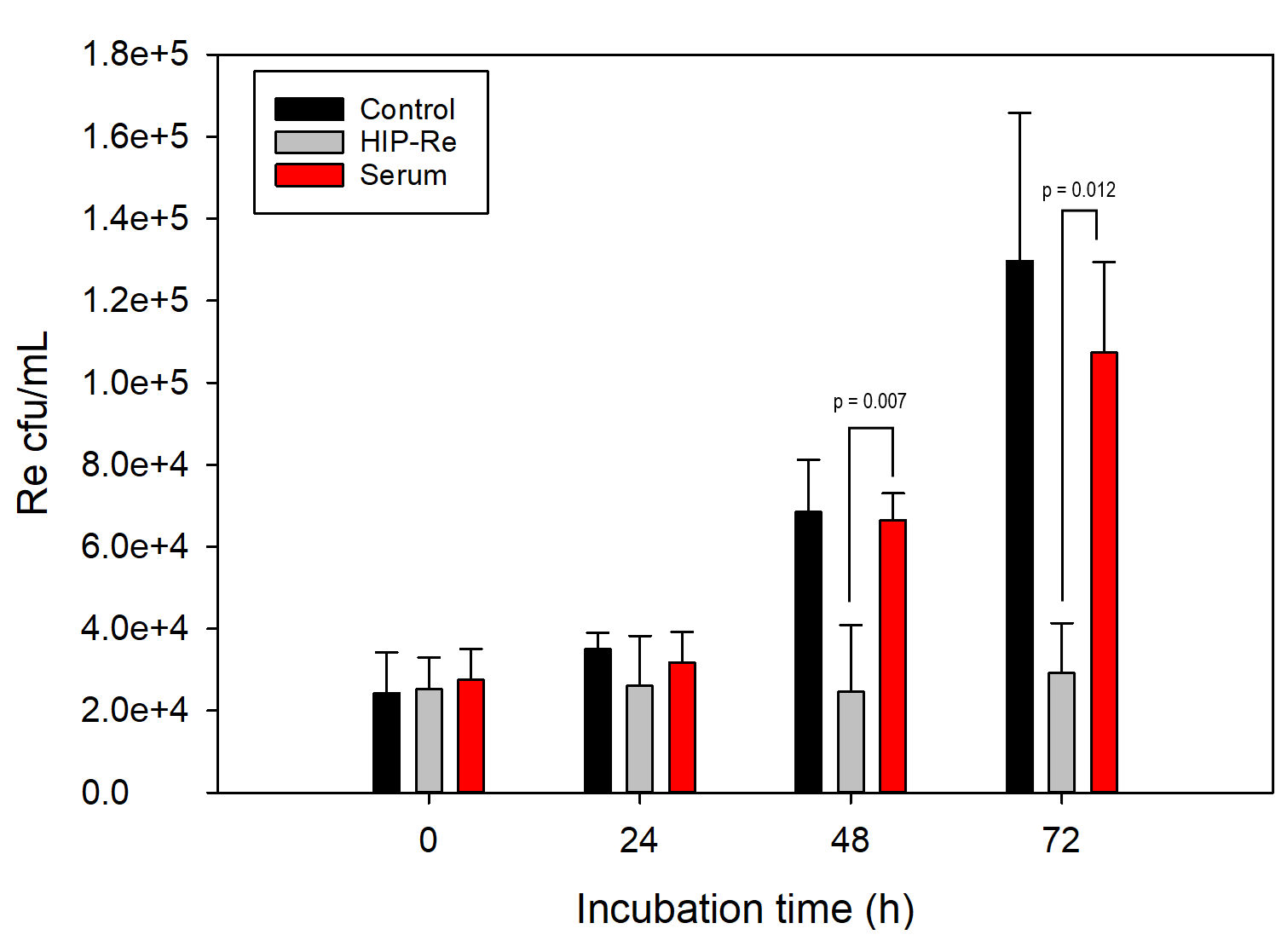


**Figure S2.** *R. equi* (log CFU/mL) after 1x10^5^ RAW264.7 cells were infected with non-opsonized (control) or opsonized with *R. equi*-specific HIP (HIP-Re) or serum *R. equi* at a MOI 20 for 1 h. Infected cells were lysed at 0, 24, 48, and 72 h post-infection, were plated in BHI agar plates and bacterial colonies were counted 48 h later. Statistical significance between groups is noted in the graph.
